# Evaluation of Variability Across Rat Acute Oral Systemic Toxicity Studies

**DOI:** 10.1093/toxsci/kfac042

**Published:** 2022-04-15

**Authors:** Agnes L Karmaus, Kamel Mansouri, Kimberly T To, Bevin Blake, Jeremy Fitzpatrick, Judy Strickland, Grace Patlewicz, David Allen, Warren Casey, Nicole Kleinstreuer

**Affiliations:** Integrated Laboratory Systems, LLC, Morrisville, North Carolina 27560, USA; National Toxicology Program Interagency Center for the Evaluation of Alternative Toxicological Methods, National Institute of Environmental Health Sciences, Research Triangle Park, North Carolina 27709, USA; Integrated Laboratory Systems, LLC, Morrisville, North Carolina 27560, USA; National Toxicology Program Interagency Center for the Evaluation of Alternative Toxicological Methods, National Institute of Environmental Health Sciences, Research Triangle Park, North Carolina 27709, USA; Center for Computational Toxicology and Exposure, Office of Research and Development, U.S. Environmental Protection Agency, Research Triangle Park, North Carolina 27711, USA; Integrated Laboratory Systems, LLC, Morrisville, North Carolina 27560, USA; Center for Computational Toxicology and Exposure, Office of Research and Development, U.S. Environmental Protection Agency, Research Triangle Park, North Carolina 27711, USA; Integrated Laboratory Systems, LLC, Morrisville, North Carolina 27560, USA; National Toxicology Program Interagency Center for the Evaluation of Alternative Toxicological Methods, National Institute of Environmental Health Sciences, Research Triangle Park, North Carolina 27709, USA; National Toxicology Program Interagency Center for the Evaluation of Alternative Toxicological Methods, National Institute of Environmental Health Sciences, Research Triangle Park, North Carolina 27709, USA

**Keywords:** acute toxicity, *in vivo* variability, LD50, establishing confidence

## Abstract

Regulatory agencies rely upon rodent *in vivo* acute oral toxicity data to determine hazard categorization, require appropriate precautionary labeling, and perform quantitative risk assessments. As the field of toxicology moves toward animal-free new approach methodologies (NAMs), there is a pressing need to develop a reliable, robust reference data set to characterize the reproducibility and inherent variability in the *in vivo* acute oral toxicity test method, which would serve to contextualize results and set expectations regarding NAM performance. Such a data set is also needed for training and evaluating computational models. To meet these needs, rat acute oral LD_50_ data from multiple databases were compiled, curated, and analyzed to characterize variability and reproducibility of results across a set of up to 2441 chemicals with multiple independent study records. Conditional probability analyses reveal that replicate studies only result in the same hazard categorization on average at 60% likelihood. Although we did not have sufficient study metadata to evaluate the impact of specific protocol components (eg, strain, age, or sex of rat, feed used, treatment vehicle, etc.), studies were assumed to follow standard test guidelines. We investigated, but could not attribute, various chemical properties as the sources of variability (ie, chemical structure, physiochemical properties, functional use). Thus, we conclude that inherent biological or protocol variability likely underlies the variance in the results. Based on the observed variability, we were able to quantify a margin of uncertainty of ±0.24 log_10_ (mg/kg) associated with discrete *in vivo* rat acute oral LD_50_ values.

Acute systemic toxicity studies are used by regulatory agencies to determine hazard categorization, assign appropriate labeling to alert consumers to potential toxicity hazards, and in risk assessment applications (Strickland *et al.*, 2018). The *in vivo* regulatory test guidelines are used to determine a dose level expected to result in 50% lethality for tested animals following a single oral administration of test substance (oral LD_50_). There are regulatory needs for experimentally derived discrete point estimates of oral LD_50_ values (eg, when used to determine acceptable exposure limits), but in other instances a range of LD_50_ values may be acceptable (eg, to define personal protective equipment requirements; Morris-Schaffer and McCoy 2021; Strickland *et al.*, 2018). To address these regulatory needs, multiple protocols have been internationally harmonized by the Organization for Economic Co-operation and Development (OECD) health effects test guidelines to identify potential acute oral toxicants (OECD, 2002a,b, 2008).

As the field of toxicology moves toward the development of new approach methodologies (NAMs) that do not require the use of laboratory animals, there is an inherent need for fully characterized reference test data against which NAMs can be compared. For acute oral toxicity, rodent LD_50_ values serve as the primary reference comparator, and therefore it is important to compile a resource of *in vivo* reference data and to characterize the variability of these values based on independently conducted studies for the same test substances. Simply put, a solid understanding of the reproducibility and inherent variability of the rat *in vivo* acute oral toxicity assays will provide a foundation to contextualize results and set expectations regarding NAM performance.

Several studies have previously evaluated the within- and between-laboratory reproducibility of the *in vivo* test method to demonstrate the impact of modifying protocol components such as rat strain, vehicle, or age of rat from a relatively small (*n* < 30) number of chemicals (Griffith, 1964; Hunter *et al.*, 1979; Weil and Wright, 1967; Weil *et al.*, 1966). Other studies compiled larger (*n* = 62–88) lists of reference chemicals with replicate LD_50_ values to establish a reported range as a measure of variability (Hoffmann *et al.*, 2010; ICCVAM, 2006). However, with the expansive list of chemicals in global commerce that include substances registered by multiple manufacturers, far greater numbers of chemicals have been evaluated for acute oral toxicity numerous times. These data have become increasingly available through publicly accessible web-based resources, thereby providing an opportunity to collate large databases of toxicology study results.

Herein, we present the largest assembly to date of manually curated rat acute oral toxicity LD_50_ data comprising chemicals with more than one experimentally derived LD_50_ value retrieved from multiple international data sources. We have accounted for data redundancy, evaluated LD_50_ distribution/variance, applied cheminformatics analyses, and defined a margin of uncertainty that can be applied when considering *in vivo* acute oral toxicity data or predictions thereof. Given the long history of regulatory decisions based upon LD_50_, establishing this margin of uncertainty will more effectively build scientific confidence in results generated by NAMs as compared to *in vivo* results.

## MATERIALS AND METHODS

###  

#### Data Sources and Inventory Compilation

Data sources for rat acute oral toxicity were selected based on data accessibility or availability and included the following:

- ChemProp (European Chemicals Agency [ECHA], https://echa.europa.eu/information-on-chemicals; last accessed November 2018)
- Hazardous Substances Data Bank (National Library of Medicine, https://www.nlm.nih.gov/databases/download/hsdb.html; last accessed November 2018)
- ChemIDplus (National Library of Medicine, https://chem.nlm.nih.gov/chemidplus/chemidlite.jsp; last accessed November 2018)
- AcutoxBase (European Union Joint Research Centre, Kinsner-Ovaskainen *et al.*, 2009)
- eChemPortal (OECD, https://www.echemportal.org/echemportal/; last accessed November 2018)

Data compilation was restricted to rat LD_50_ values, retaining only LD_50_ values that were amenable to conversion into mg/kg units. Where unit conversion was required, molecular weights were retrieved from the U.S. Environmental Protection Agency (EPA) CompTox Chemicals Dashboard (Williams *et al.*, 2017; https://comptox.epa.gov/dashboard; last accessed November 2018) using Chemical Abstracts Service Registry Numbers (CASRN) as input. In addition to species and LD_50_ unit filtering, data were also manually curated (described below). Compiled LD_50_ values were in the form of point estimates, limit tests, and acute toxic class ranges (OECD, 2002b).

The data set was compiled using CASRN as the primary chemical identifier. When more than one CASRN mapped to the same chemical structure (including deprecated CASRNs), these data entries were not collapsed/corrected, but rather kept separate to match the identifier used in the database of origin and to reflect unique experimental records; thus, unique chemical counts reflect unique CASRN, not necessarily unique chemical structures.

The initial compilation yielded a total of 15 688 unique chemicals with at least one rat LD_50_ value. The inclusion of multiple instances for the same LD_50_ value for any given chemical was limited to avoid overrepresenting studies that may have been reported in multiple data sources. A subset of chemicals for which at least 2 unique discrete point estimate LD_50_ values were available was selected and manually curated to create the foundation of the inventory used for the current study. Subsequently, data generously provided directly by ECHA were added to yield a final data set of 1885 chemicals and 5826 quantitative LD_50_ values (provided in Supplementary File 1). Additional acute oral systemic toxicity data that were not reported as discrete point estimate LD_50_ values, but rather as limit tests or acute toxic class ranges, were also retrieved and added to the discrete LD_50_ values to generate an expanded inventory for further hazard category-based analyses. When these values were added to the discrete LD_50_ data set, the expanded categorical data set contained 7574 entries representing 2441 chemicals (also provided in Supplementary File 1). This data set retained the requirement that chemicals have at least 2 unique entries.

Additional physicochemical data for various analyses conducted in this project were retrieved from a variety of sources. Chemical structures and properties were retrieved from the EPA Comptox Chemicals Dashboard (Williams *et al.*, 2017) and generated by the Open Structure-activity/property Relationship App (OPERA; Mansouri *et al.*, 2018). We used simplified molecular-input line-entry system (SMILES) strings for structure-related analyses. These were retrieved from the EPA CompTox Chemicals Dashboard with all queries conducted using CASRNs as input. ToxPrint (ChemoTyper v1.0; Yang *et al.*, 2015) chemotypes fingerprints were used for cheminformatics assessments. Finally, product use information for each chemical was obtained from the EPA Consumer Product Categories (CPCat) database (Dionisio *et al.*, 2018) to characterize the diversity of the data set and evaluate any trends associated with variability.

#### Curation of the Rat Acute Oral Systemic Toxicity Inventory

Curation of the compiled data set involved identifying unique LD_50_ values per CASRN in order to omit values that may have been the same data point present in multiple source databases to avoid overrepresentation. For example, a chemical might have 3 LD_50_ values of “>2000 mg/kg” based on limit test results. Because the majority of databases we used did not include primary source references for the original studies, it was impossible to determine whether this data point originated from 3 independent studies or arose from one study that was captured in multiple source databases. As such, only one value of >2000 mg/kg was kept in the final data set. Where available, qualifiers associated with limit tests were noted and tracked. For example, “>” or “<”, reflecting “greater than” or “less than” designations for limit test outcomes, respectively, were noted.

We removed each of the following: values derived from complex mixtures reported as individual substances; values from studies having an “unreported” exposure route (the required exposure route was oral); and values exceeding 10 000 mg/kg because they were considered to be unrealistically high. Data reported as ranges or sourced from acute toxic class or limit tests were separated into a unique inventory. These values were integrated with the main data set only for categorical analyses, where EPA or United Nations Globally Harmonized System of Classification and Labelling of Chemicals (GHS) hazard categories could be assigned despite the lack of discrete point estimate LD_50_ values.

Based on expert judgment, manual curation removed any data identified as potentially erroneous. Such values were traced back to their source database to determine if they could be confirmed. For example, in some instances, 3 values for the same substance were reported that were both similar and very precise (eg, 22.2, 23.6, and 25). Upon further inspection, it was determined that these values were actually the mean and 95% confidence interval for that substance; in these cases only the mean value was retained in our data set and the other 2 values were excluded. In another example, 3 values were reported for the same substance that, upon further inspection, were determined to be separate values calculated based on either males, females, or a mean value calculated from both sexes; in these cases only one value was retained. In yet another example, multiple LD_50_ values for the same substance were flagged because they were the same as those associated with LD_50_ ranges used for hazard classification (eg, 300 mg/kg, 2000 mg/kg). The study metadata revealed that the results were actually a reported LD_50_ range (300–2000 mg/kg) generated from the acute toxic class protocol that was mistakenly recorded as 2 separate LD_50_ values, these data were kept only in the expanded data set.

#### Additional Systematic Evaluation for Possible Read-Across Data

The lack of metadata available for the retrieved data compiled herein created another source of uncertainty around the acute oral toxicity values. For example, some LD_50_ values were reported as experimental point estimate values, but manual curation identified the original toxicity assessment report to be based on a read-across analysis from an analog structure (see ECHA dossier on 3-methylpentane, https://www.echa.europa.eu/web/guest/registration-dossier/-/registered-dossier/24591/7/3/2; last accessed November 2018). Once this was discovered, we used cheminformatics tools to try to identify other instances where data points may have originated from read-across. For this, we tested 2 types of hypotheses for which the findings are described below. Results suggest that some of the data points in our compiled inventory may have originated from read-across studies. However, without clear metadata, as was the case for 3-methylpentane, it was not possible to prove with certainty that even the most similar chemicals are a result of a read-across.

##### Read-across analysis 1: Based on association of multiple CASRNs with the same set of LD_50_ values

We identified chemical clusters where multiple CASRNs were associated with the same set of LD_50_ values. In total, there are 47 clusters of chemicals sharing at least 2 LD_50_ values. The clusters were identified using the CASRNs matched to the same Distributed Structure-Searchable Toxicity Database identifiers (DTXSIDs; Grulke, 2019), names, original structures, and quantitative structure-activity relationship (QSAR)-ready structures (Supplemental File 2, “repeated_LD50.csv”; Mansouri, 2016). Upon evaluating these clusters of chemicals, we identified several cases of single chemicals with multiple CASRNs or different salts like boric acid and calcium borate, silver sulfate and silver chloride, sodium lactate and calcium lactate, magnesium vanadate (MgV_2_O_6_), and calcium vanadate (Ca(VO_3_)_2_), all of which would generally have resulted in the same QSAR-ready structure, and had the same set of LD_50_ values. We also found instances of chemicals that exist in different forms, like d-dilactide and 3,6-dimethyl-2,5-dioxo-1,4-dioxane, camphor and d-camphor, or citronellal and (3R)-3,7-dimethyloct-6-enal. Finally, there were cases where 2 different CASRNs represented different forms of a chemical that can coexist in the same solution, and the LD_50_ was assigned to both, such as *p*-xylene and *m*-xylene, or cinerin I and cinerin II.

On the other hand, we concluded that the equivalent LD_50_ values of ethane-1,2-diyl bis(sulfanylacetate) and 2-ethylhexyl thioglycolate were derived from independent studies, as the structures of these 2 substance looked too different for the values to have been generated by read-across. Similarly, although chloroxuron and dinitramine had very similar structures, their LD_50_ values (3000 and 3700 mg/kg, respectively) seemed more like limit test values, despite having been recorded as point estimates in the source databases. In conclusion, despite these chemical clusters appearing to have the exact same data, our review suggested that none were conclusively the result of read-across and could not be excluded from the compiled inventory for that reason.

##### Read-across analysis 2: Based on structural similarity

This analysis approach targeted chemical structures and compared them based on multiple chemicals sharing the same single LD_50_ (as opposed to groups of CASRNs sharing multiple LD_50_s, examined in the previous analysis). First, groups were formed by identifying CASRNs sharing a single LD_50_. Then, extended fingerprints generated using CDK were used to evaluate similarity using pairwise Tanimoto indices, see Supplemental File 3 (repeated_LD50_analogs.xlsx). For example, 6 CASRNs in the compiled inventory had an LD_50_ of 2 mg/kg. After generating the similarity matrix of the 6 chemicals, we pulled those with Tanimoto similarity scores between 0.7 and 0.95, representing the range of structures that are similar enough for read-across analysis but not actually the same structure. Out of the 6, only 2 were within the similarity range for read-across (0.9 in this example). We identified 41 such groups, some of which were likely candidates for read-across given the chemical similarity scores. However, there were no cases where we were able to definitively identify LD_50_ data points that were generated by read-across. Furthermore, out of many structures with the same LD_50_ values, only 2 groups of chemicals were in the appropriate similarity range, with the remaining structure too dissimilar to suspect read-across. Thus, if some substances had the same LD_50_ and were very different structurally, we cannot make the case that only the identical LD_50_ values from chemicals with similar structures resulted from read-across and should potentially be excluded. This possibility, however, is worth noting as an additional source of uncertainty in the data set that we have attempted to quantify.

#### Analysis Approaches

All analyses were conducted in R (version 3.6.0) unless stated otherwise, and all these analyses and figure generation code are provided in an R script in Supplementary File 4. A representative LD_50_ for each chemical was computed as the median of only replicate point estimate LD_50_ values (ie, not including limit tests or hazard categorical data). Similarly, variability was computed as the median absolute deviation (MAD) across log_10_ of the point estimate LD_50_ values only. The correlation between each of the experimental LD_50_ point estimate values and the chemical-specific median LD_50_ was assessed using Pearson’s correlation *r*^2^ and root mean-squared error (RMSE). Chemical-specific MADs were evaluated relative to chemical potency, number of replicate studies, and hazard categories. A global margin of error around the median LD_50_ was computed as ±2.5×MAD, as recommended for outlier detection (Leys *et al.*, 2013). To assess the likelihood of hazard category concordance across repeat studies, conditional probabilities were calculated as described in Luechtefeld *et al.* (2016). The probability of a chemical being in category *j* (*C*_2_ = *j*) given that it has been previously categorized in category *i* (*C*_1_ = *i*) is calculated as:

$$P\left(C_{2}=j|C_{1}=i\right)=\frac{n_{\left(i\right)j}}{n_{\left(i\right)}}$$

where *i* and *j* are hazard categories, *n*_(__*i*__)_ is the number of studies for chemicals classified in category *i* at least once, and *n*_(__*i*__)__*j*_ is the number of studies within *n*_(__*i*__)_ where a chemical was classified in category *j*.

Chemical use categories, structural descriptors, toxicity-related fingerprints, and physicochemical properties were examined for trends associated with LD_50_ variability. MATLAB (version 9.4) was used for categorical analyses for concordance, ToxPrint, and physiochemical property analyses.

## RESULTS

###  

#### Rat Acute Oral Systemic Toxicity Inventory

**Figure 1.**
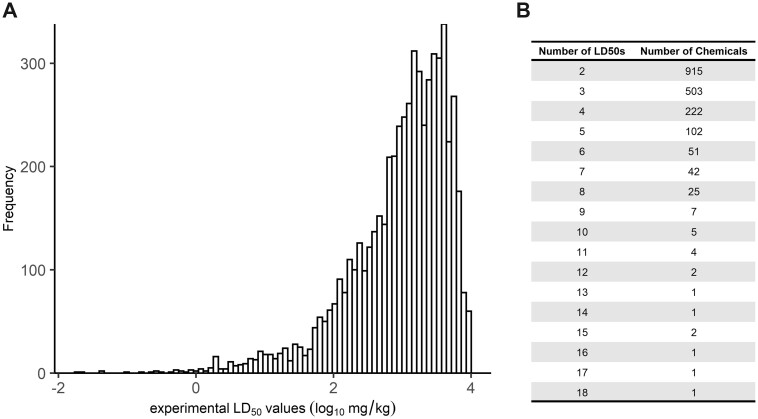
Characterizing the LD_50_ data set. The curated rat acute oral systemic toxicity LD_50_ data set comprised chemicals with at least 2 point estimate LD_50_ values, resulting in an inventory of 5826 LD_50_ values representing 1885 chemicals. A, Histogram of the distribution of LD_50_ values in the data set. B, Summary of replicate LD_50_ values in the data set per chemical. The LD_50_ values ranged from 0.02 to 10 000 mg/kg, with most values between 1000 and 10 000 mg/kg as shown in the histogram. Most chemicals (1742/1885 chemicals) had 5 or fewer LD_50_ values.

A data set of rat acute oral LD_50_ values, for chemicals with at least 2 independent experimentally derived discrete point estimate values, was compiled comprising 1885 chemicals and 5826 LD_50_ values. Figure 1A shows the distribution of these point estimate LD_50_ values, revealing that the 5826 LD_50_ values in the data set ranged from 0.02  to 10 000 mg/kg. Most values were above 1000 mg/kg (3349/5826 values, ie, 57.5%). Additionally, the number of unique LD_50_ values per chemical was assessed (Figure 1B) revealing that the majority of chemicals had 2 (915 chemicals) or 3 (503 chemicals) unique point estimate LD_50_ values. There were 18 chemicals with 10 or more point estimate LD_50_ values in the data set.

#### Characterizing Acute Oral Toxicity Study Reproducibility and Variability

**Figure 2.**
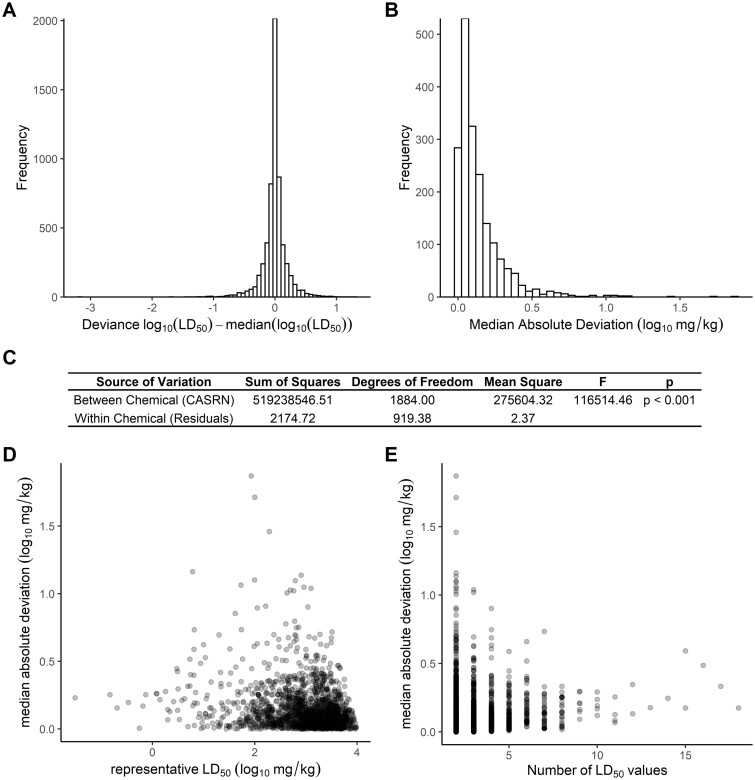
Evaluating variability of LD_50_ replicates. For each of the 1885 chemicals with at least 2 discrete point estimate LD_50_ values, a median value was used as the chemical-specific representative LD_50_. Deviation was derived by subtracting the chemical median from the individual LD_50_ values (A). MAD values computed per chemical are plotted as a histogram to visualize frequency of values (B). Welch’s ANOVA table (C) summarizes statistical analyses that confirm most variability is attributable to interchemical (between chemicals) rather than intrachemical (within chemical) variability. Variability among replicate point estimate LD_50_ values was assessed by calculating MAD across replicate LD_50_ values per chemical in log_10_ mg/kg units. Plots show MAD values as a function of chemical potency (D) or the number of replicate LD_50_ values available per chemical (E).

To assess reproducibility of discrete LD_50_ point estimates from replicate studies for a given chemical, a single representative LD_50_ value was calculated for each chemical to serve as a reference value. Given increased confidence due to manual curation, representative LD_50_ values were defined per chemical as the median of all point estimate LD_50_ values for a single chemical. *In vivo* study reproducibility was then assessed as a measure of how each experimentally derived LD_50_ value (at least 2 independent experiments per chemical) compared with the representative value (used as “truth”). Most of the 5826 experimental LD_50_ point estimate values were within one order of magnitude from the chemical-specific median LD_50_ value (representative value; Figure 2A). This plot visualizing the deviation from the median shows a peak at 0 where uneven replicate LD_50_ values resulted in the median being exactly one of the experimental values. The high Pearson’s correlation (*r*^2^ of 0.927) and low RMSE (0.197) confirm the appropriateness of the median LD_50_ representative value for the experimental data set.

To quantify the variability, MAD was calculated for each chemical using discrete point estimate experimental LD_50_ values. The distribution of MAD (Figure 2B) reveals that most MAD values are below 0.5, though there are a few chemicals with high MAD values, the top10 most variable chemicals based on MAD are listed in Table 1. Iridium tetrachloride (CASRN 10025-97-5) had the highest MAD of 1.87 (log_10_ (mg/kg)), based on 2 independent LD_50_ point estimate values that were 3 orders of magnitude apart: 4.67 mg/kg and 1560 mg/kg. The median MAD across all 1885 chemicals was 0.0895 log_10_ (mg/kg).

**Table 1.** Top 10 Most Variable Chemicals Based on Median Absolute Deviation

CASRN	Chemical Name	Median Absolute Deviation (log_10_ (mg/kg))	Number of LD_50_ Values	LD_50_ Values (mg/kg)
10025-97-5	Iridium tetrachloride	1.871	2	4.67, 1560
97-18-7	Bithionol	1.713	2	7, 1430
62-56-6	Thiourea	1.459	2	20, 1860
7487-94-7	Mercuric chloride	1.163	2	1, 37
104-12-1	4-Chlorophenyl isocyanate	1.137	2	138, 4710
7719-12-2	Phosphorous trichloride	1.101	2	18, 550
72-54-8	*p*,*p*'-DDD	1.096	2	113, 3400
83-26-1	Pindone	1.063	2	10.3, 280
2374-14-3	2,4,6-Trimethyl-2,4,6-tris(3,3,3-trifluoropropyl)cyclotrisiloxane	1.047	2	180, 4659
117-18-0	2,3,5,6-Tetrachloronitrobenzene	1.039	3	250, 1256, 7500

The variability of LD_50_ values was significantly different between chemicals (Brown-Forsythe *p* < .001). To confirm that interchemical variability was the primary source of variance rather than intrachemical replicate variability, a Welch’s ANOVA was performed as this approach does not assume equal variances and adjusts for differing group sizes in the derivation of the residual degrees of freedom (Welch, 1951). The Welch’s ANOVA confirmed that in the linear model between log10 LD_50_ and chemical, most variability is attributable to inter-chemical variability (Figure 2C).

To assess whether any trends existed between variability and either the number of studies for a chemical or the chemical’s potency (eg, LD_50_), we evaluated the chemical-specific MADs relative to both of these factors (Figure 2D and 2E). Most chemicals had a relatively low MAD (represented by the *y*-axis spread of Figures 2D and 2E). We found that the MADs had low correlation with chemical potency (linear trend *r*^2^ = 0.046) and that MAD did not increase or decrease as the number of study replicates increased (Jonckheere-Terpstra test *p *>* *.05). It should, however, be noted that the sample distributions were rather biased, with relatively few chemicals having a high number of repeated studies. Thus, we suggest that variability, represented as high MADs over multiple studies, may be associated either with potency or with the number of replicate studies available. In general, it is difficult to conclude that the variability observed was simply due the number of times the rat acute oral toxicity assay was conducted.

#### Impact of Variability on Hazard Categorization

**Figure 3.**
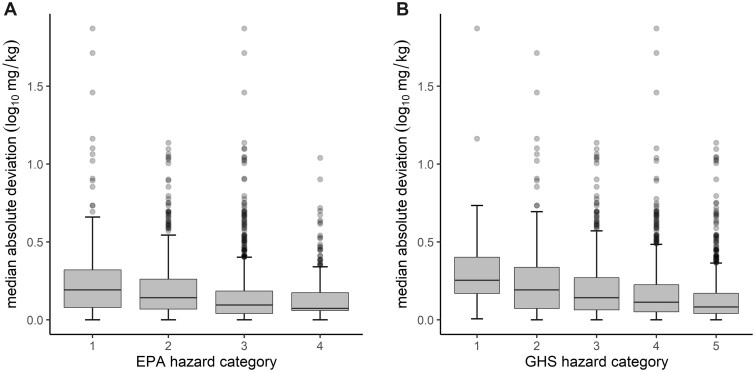
MAD of LD_50_ values per chemical in each hazard category. The MAD (log_10_ mg/kg units) across all LD_50_ values, per chemical, was computed for each of the 1885 chemicals having at least 2 independently reported LD_50_ values. The boxplots reflect the distribution of MADs for each chemical classified into each respective hazard category for EPA categorization (A) and GHS categorization (B) schema, respectively, where lower hazard category numbers correspond to lower/more potent LD_50_ values. These distributions demonstrate that MAD, as a measure of variability, may be correlated with hazard category (ie, more potently toxic chemicals have higher variability; Jonckheere-Terpstra trend test *p* value = .0002 for both classification systems).

Acute oral toxicity studies are frequently used to derive hazard categorizations for safety labeling. Accordingly, we mapped the chemical-specific representative point estimate LD_50_ values to hazard categories using both the EPA (EPA, 2018) and GHS (UN, 2021) hazard classification systems. The association between hazard categories (ie, potency) and quantitative variability for the 1885 chemicals was evaluated by generating boxplots of the chemical-specific MADs for all chemicals per hazard category (Figure 3). A generally decreasing median was observed in MAD distributions across hazard categories, which were significant as tested by a one-sided Jonckheere-Terpstra trend test (*p* value .0002 for both the EPA and GHS systems; Figure 3A and 3B).

When limit tests and acute toxic class range values were added to the discrete LD_50_ data set, the expanded categorical data set contained 7574 entries representing 2441 chemicals. Note that the categorical data set encompassed the entirety of the discrete point estimate LD_50_ values (5826 entries representing 1885 chemicals). Because not all data points in this categorical data set were discrete values, a median or MAD per chemical could not be calculated for evaluating reproducibility or quantifying variability by hazard category. Instead, chemicals were grouped based the number of hazard categories they could be assigned to using the available replicate study data.

Concordance across hazard category determinations was computed and evaluated as compared with the number of experimental replicates and number of entries per hazard category (Supplementary Figure 1), revealing that increased study replication does not correlate with category concordance. In fact, disparate hazard categorization (ie, chemicals mapping to more than one hazard category) was seen for nearly 40% of chemicals with either classification schema. For example, of the 2441 chemicals in the expanded categorical data set, 809 were mapped to more than one EPA hazard category and 969 were mapped to more than one GHS category. The chemicals with the most variable LD_50_ values were associated with as many as 3 EPA hazard categories (19 chemicals) or with 3 (33 chemicals) and even 4 (2 chemicals) GHS hazard categories. The majority of data points in the expanded categorical acute oral toxicity data set were associated with EPA hazard Category III or GHS Categories 4 and 5 (Table 2), reflecting the general bias of the data set toward less potent chemicals for hazard category evaluation.

**Table 2.** Hazard Categorization of Expanded Inventory

EPA Category	GHS Category	Number of LD_50_ Entries^a^	Number of Chemicals^a^
I (LD_50_ ≤ 50 mg/kg)	1 (LD_50_ ≤ 5 mg/kg)	104	53
I (LD_50_ ≤ 50 mg/kg)	2 (5 < LD_50_ ≤ 50 mg/kg)	342	183
II (50 < LD_50_ ≤ 500 mg/kg)	3 (50 < LD_50_ ≤ 300 mg/kg)	1166	556
II (50 < LD_50_ ≤ 500 mg/kg)	4 (300 < LD_50_ ≤ 2000 mg/kg)	528	354
III (500 < LD_50_ ≤ 5000 mg/kg)	4 (300 < LD_50_ ≤ 2000 mg/kg)	2567	1309
III (500 < LD_50_ ≤ 5000 mg/kg)	5 (LD_50_ > 2000 mg/kg)	2079	1032
IV (LD_50_ > 5000 mg/kg)	5 (LD_50_ > 2000 mg/kg)	788	458

a Note that although the sum of LD_50_ entries equals the total number of chemicals in the inventory (7574 entries), the total number of chemicals does not equal the sum of unique chemicals in the inventory (2441 chemicals) because many chemicals have entries (eg, LD_50_ values) in more than one row depending on whether replicate study results mapped to different categories.

The impact of variability in hazard category assignment across independently conducted replicate acute oral toxicity studies was summarized by conditional probabilities analysis. Briefly, conditional probabilities are a measure of the probability that, upon repeat testing, a chemical will be categorized into a given hazard category once a prior study has already categorized the chemical. This approach has similarly been applied to ocular and dermal toxicity studies to evaluate the reproducibility of these in vivo methods in identifying hazard categories (Luechtefeld *et al.*, 2016; Rooney *et al.*, 2021). Using the expanded categorical data set, the conditional probabilities were computed across the experimental data and summarized for EPA hazard categorization (Table 3) and GHS categorization (Table 4). For the EPA hazard system, an assignment to Category III was most likely to be replicated by subsequent studies, with a probability of 79.8%, whereas replication of a Category IV outcome was only 54.6%. Similarly striking results were observed for the GHS schema where an assignment to category 5 was most likely to be reproduced with the highest probability being 75%. These results suggest that reproducibility of the rat acute oral toxicity study for hazard categorization is closer to 50%–60% as studies are replicated and not nearly as consistent as might be expected.

**Table 3.** Conditional Probabilities for EPA Hazard Category Reproducibility

		Conditional Probability of Subsequent Study Categorization			
		I	II	III	IV
First Study Hazard Category	**I**	**57.9%**	34.5%	6.2%	1.3%
	**II**	5.7%	**66.5%**	27.5%	0.4%
	**III**	0.5%	11%	**79.8%**	8.7%
	**IV**	0.1%	0.6	44.7%	**54.6%**

Bold values represent conditional probability of subsequent studies identifying the same hazard category as the first study.

**Table 4.** Conditional Probabilities for GHS Hazard Category Reproducibility

		Conditional Probability of Subsequent Study Categorization				
		1	2	3	4	5
First Study Hazard Category	**1**	**53.3%**	34.9%	1.5%	5.1%	5.1%
	**2**	7.7%	**48.9%**	33.2%	8.9%	1.3%
	**3**	0.2%	7.1%	**61.9%**	28.9%	1.9%
	**4**	0.1%	1%	11.0%	**66.1%**	21.8%
	**5**	0%	0.2%	1%	23.8%	**75%**

Bold values represent conditional probability of subsequent studies identifying the same hazard category as the first study.

#### Evaluating Possible Chemical Sources of Variability

**Figure 4.**
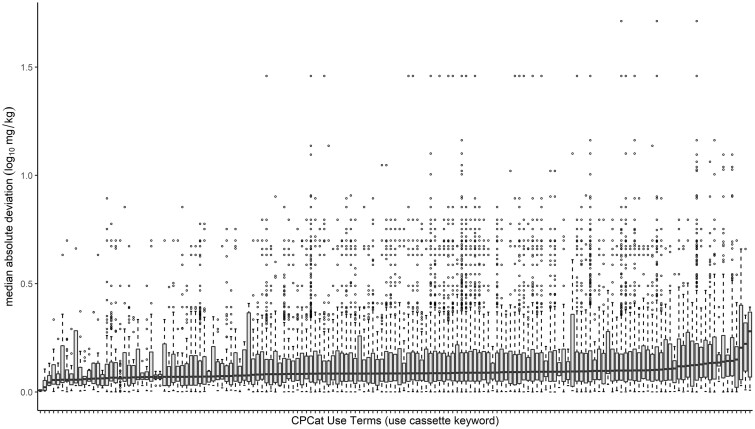
Evaluation of LD_50_ median absolute deviation (MAD) per chemical use category. The 1619 chemicals with at least 2 discrete point estimate LD_50_ values could be mapped to chemical use categories in CPCat. Chemicals were associated with anywhere from 1 to 128 use terms among 181 unique use categories present in the data set. The 161 use category terms having at least 3 chemicals mapped are all represented across this figure (ie, each respective boxplot along the *x*-axis). The boxplots represent the distribution of LD_50_ MADs (in log_10_ mg/kg units) for the chemicals associated with each use term, respectively. Although the MAD across the chemicals mapped to each use term increases from left to right across the plot, the range of MADs (boxplots) generally overlap, confirming that there is no use term with significantly lower/higher variable chemicals.

An investigation to assess whether chemical use was correlated with variability was also conducted. Chemical use categories were retrieved from the CPCat database (Dionisio *et al.*, 2018). Of the 1885 chemicals with discrete point estimate LD_50_ values, 1619 had chemical use information. There were 181 unique use terms represented across the 1619 chemicals with retrieved use information. Chemicals were often associated with more than one use term; in fact, the number of use terms per chemical ranged from one to 128. Similarly, we also assessed how many chemicals were associated with each use term present in the data set, revealing a range of 1 to 1093 chemicals being associated with any term. There were 5 use terms associated with over 500 chemicals: *manufacturing* (1093 chemicals), *industrial manufacturing* (1048 chemicals), *pesticide* (901 chemicals), *consumer use* (713 chemicals), and *personal care* (518 chemicals). This analysis was repeated with the expanded categorical data set, yielding the same results (data not shown). To evaluate whether variability was associated with use terms, we focused on 161 use terms with at least 3 associated chemicals (Figure 4). An examination of this data subset revealed that the range of LD_50_ variability was comparable for all use terms represented in the data set. The lowest median MAD category (left-most boxplot in Figure 4) was *UV stabilizer*, which mapped to 3 chemicals having a combined median MAD of 0.0081 log_10_ (mg/kg). Conversely, the highest median MAD was 0.280 log_10_ (mg/kg) for the use term *friction agent*. There was no use term enriched with chemicals with more variable MADs, suggesting that use category is not correlated with variability in acute oral toxicity studies (Brown-Forsythe *p* = .27).

**Figure 5.**
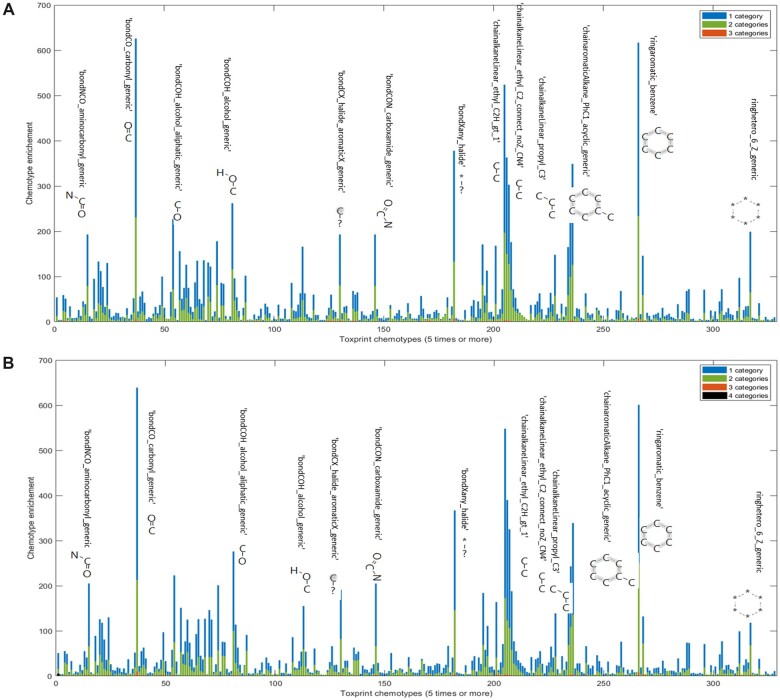
Evaluation of LD_50_ variability per ToxPrint chemotype. The expanded categorical inventory of 2441 chemicals was mapped to ToxPrint chemotypes. Chemicals were associated with 408 ToxPrint chemotypes represented, of which 224 ToxPrint chemotypes had at least 5 chemicals mapped and are represented in this figure. Evaluations for both EPA hazard category (A) and GHS hazard category (B) schema yielded comparable results in that enrichment of ToxPrint chemotypes were proportional to the number of chemicals per variability class, rather than the class itself (1 category being low variability class, 2 being moderate, and 3–4 categories being high variability class).

To investigate whether chemical structure was associated with an enrichment of greater variability in acute oral LD_50_ data, the publicly available set of 729 ToxPrint chemotypes (Yang *et al.*, 2015) were utilized to define chemicals in terms of structural features. This set of chemical fingerprints was selected because it was developed specifically with toxicology and annotation of motifs associated with mechanisms of toxicity in mind. The ToxPrint chemotypes encompass 3 sets of substructures, all of which were included for our analysis: generic structural fragments, Ashby-Tennant genotoxic carcinogen rules, and cancer threshold of toxicological concern (TTC) categories. All 2441 chemicals in our expanded categorical data set were included in this analysis. The data set chemicals were represented by 408 of the 729 total ToxPrint chemotypes, suggesting a somewhat limited chemical space compared with all possible annotations available. Of the 408 ToxPrint chemotypes represented, 224 ToxPrint chemotypes had at least 5 chemicals mapped and were included for variability assessment (Figure 5). This analysis revealed that enrichment of any ToxPrint chemotype was proportional to the number of chemicals per variability class (classes were defined based on the number of hazard categories that the chemical was mapped to, as defined in the figure legend) and that there were no significant differentiators (ie, ToxPrint substructure/fragment) associated with higher or lower LD_50_ variability, as defined by the number of categories to which a chemical was classified across experimental replicates.

**Figure 6.**
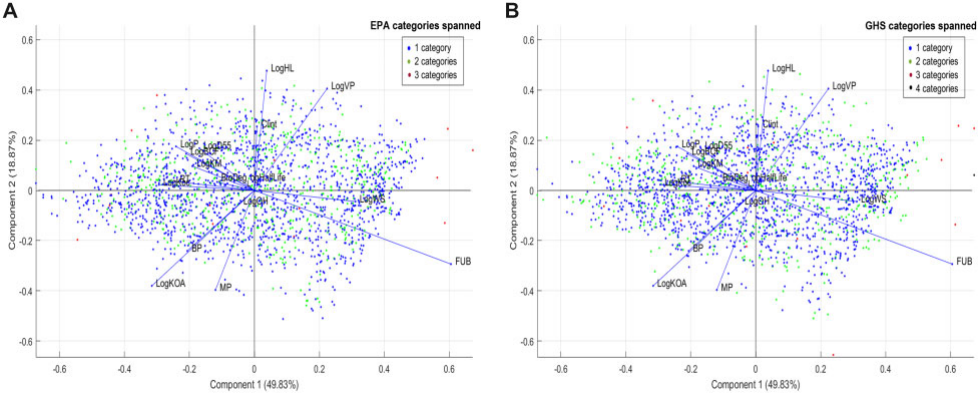
Evaluation of LD_50_ variability and physiochemical properties. The physiochemical properties for the expanded categorical inventory of 2441 chemicals, grouped into the same variability class groupings as Figure 7 (1 category being low variability class, 2 being moderate, and 3–4 categories being high variability) were retrieved from OPERA. PCA was conducted for both EPA hazard category (A) and GHS hazard category (B) schema yielded comparable results. Overall, there was no discernable physiochemical property related to variability.

To further explore factors that may have affected variability, physicochemical properties for the 2441 chemicals in the expanded categorical data set were retrieved from OPERA and evaluated using principal component analysis (PCA) for any association with variability, as measured by the number of hazard categories that each chemical was classified to based on replicate LD_50_ study data. There were 17 physiochemical properties included in the analysis, including melting point, boiling point, and solubility properties. The PCA analysis yielded comparable results whether considering EPA or GHS categorization (Figure 6), with no clear clustering or trend for any physiochemical property, confirming that physiochemical properties were not correlated with variability in this data set of acute oral LD_50_ values.

#### Defining a Margin of Uncertainty for Interpreting LD_50_ Data

Given the variability documented herein for chemicals that have been evaluated in several independently conducted rat acute oral systemic toxicity studies and the impact on hazard categorization, it is apparent that some degree of uncertainty should be associated with experimentally derived LD_50_ values. In an effort to determine a margin of uncertainty based on experimental LD_50_ values, we leveraged our inventory of discrete point estimate rat acute oral LD_50_ values. MADs for the 1885 chemicals in the data set with 2 or more point estimate LD_50_ values were bootstrapped 5000 times, and the result provided a representative MAD (0.0953 ± 0.002 log_10_ (mg/kg)) that takes into account the range of MADs in the data set. A margin of ±2.5 × MAD was computed as ±0.24 log_10_ (mg/kg), providing a moderately conservative margin of uncertainty for acute oral LD_50_ values (Leys *et al.*, 2013).

**Figure 7.**
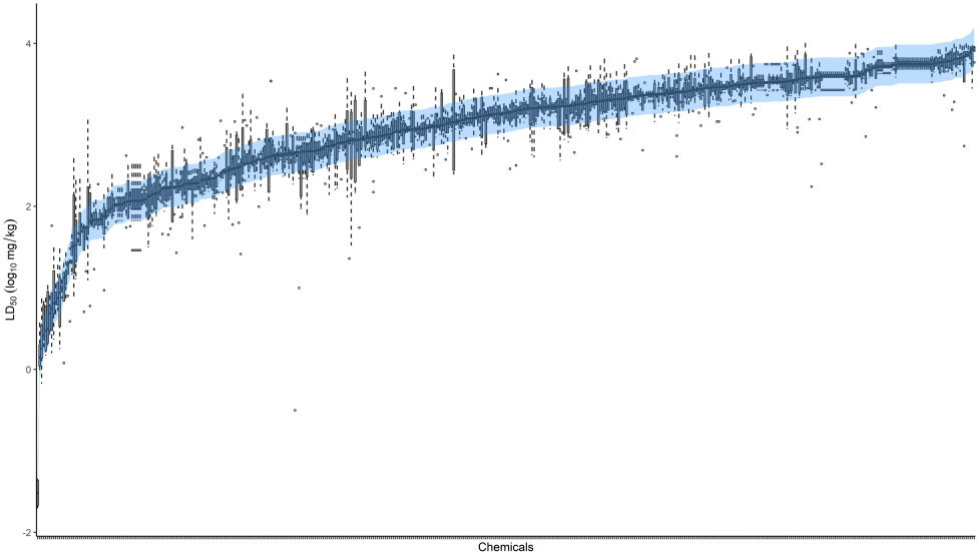
Defining an acute oral toxicity LD_50_ margin of uncertainty. For illustration purposes, only chemicals with at least 4 LD_50_ values (467 chemicals) are shown in this plot. Each chemical has a boxplot wherein the box limits represent the 25th and 75th percentiles of the data, dashed lines represent the bounds for outlier detection defined by the 1.5 interquartile range rule, and circles indicate outliers. The MADs across point estimates, per chemical, were used as input for bootstrapping (sampling 5000 times), from which the median was used to compute a margin of uncertainty. This interval equates to ±0.24 (in log_10_ mg/kg units) and is shown centered around the median of LD_50_ values per chemical (shaded area). The defined range generally encompasses the distribution of experimental LD_50_ values and serves as a reasonable range for evaluating acceptable LD_50_ estimates per chemical.

To demonstrate the range (±0.24 log_10_ (mg/kg)) against the experimental data, we highlight the uncertainty region surrounding the median LD_50_ in Figure 7. For chemicals with relatively consistent LD_50_ values across independent studies, the highlighted region encompasses all experimental LD_50_ values (represented by the boxplot). However, for those chemicals with a larger range of LD_50_ values (and higher variability), the highlighted region helps narrow down a high confidence range for each chemical’s LD_50_ estimate. For instance, converting the values back to linear range, which is consistent with experimental practice and application to classification schema, a highly toxic chemical with an LD_50_ of 5 mg/kg would have a confidence range of 2.9 to 8.7 mg/kg and for a low toxicity chemical with an LD_50_ of 5000 mg/kg it would be 2877–8689 mg/kg.

## DISCUSSION

The current study presents the most diverse and comprehensive compilation of curated rat acute oral LD_50_ data based on the consolidation of multiple resources, as well as providing broad coverage in terms of chemical structure and usage. The internationally sourced LD_50_ data contained instances of redundancy, which were limited to unique values per chemical through manual curation efforts. The compiled data allowed for comprehensive characterization of the variability and performance for the rat acute oral toxicity study. The data set is available for download via the National Toxicology Program’s Integrated Chemical Environment (https://ice.ntp.niehs.nih.gov/; last accessed January 2022).

The original data compilation, prior to the manual curation efforts undertaken here, served as the basis for several predictive modeling efforts, including the Collaborative Acute Toxicity Modeling Suite (CATMoS) collaboration (Mansouri *et al.*, 2021), in which an appropriate representative LD_50_ value was calculated for each compound and data were used to develop a consensus model for predicting rat acute oral LD_50_ (Bercu *et al.*, 2021; Borba *et al.*, 2020; Helman *et al.*, 2019; Kim *et al.*, 2020; Lunghini *et al.*, 2019). However, for the purpose of evaluating variability among replicate study results, additional data curation was conducted to increase confidence and fidelity in subsequent analyses. Our curation processes revealed several data cleaning challenges that should be considered in future data set compilation efforts. First, many values obtained from online resources overrepresented the actual number of studies conducted. For example, there were several instances where 3 LD_50_ values were extracted from the online resource, but upon closer inspection, those values represented one LD_50_ and the upper and lower bounds of its associated 95% confidence interval from a single study. Similarly, we noted multiple examples where 3 LD_50_ values were extracted, but they were all obtained from the same study and represented an LD_50_ for males, females, and both sexes combined. Second, some “extreme” LD_50_ values were chemical specific, and sometimes associated with chemicals that have multiple isomers that mapped to the same CASRN, which could introduce variability as different individual studies may have been testing different isomers. Furthermore, “extreme” LD_50_ values were also identified as being the result of unit transcription errors, for example, where mg/kg was recorded as g/kg. We also observed instances where a reported range of LD_50_ values was extracted as a single value, thereby creating a nonsensical “extreme” LD_50_ value, (eg, 500–2000 mg/kg recorded as 5 002 000 mg/kg). Collectively, these examples point to the critical need to carefully curate and review information from computationally derived data sets. However, it is important to note that there were many manually curated chemicals for which the high variability across multiple LD_50_ values could not be attributed to errors in the extraction process, and thus all seemingly reliable values were reported as experimental values herein.

Using the curated data set, analyses of data-rich chemicals (those having multiple unique LD_50_ values) revealed that independent experimental studies can yield LD_50_ values orders of magnitude apart. The variability among LD_50_ estimates for a single compound bears significant implications in regulatory applications due to disparities in hazard classification and labeling. We interrogated every source of variability that could be attributed to the chemicals themselves. We compared a wide range of parameters across the entire chemical space to determine if there were any distinct between “high variability” chemicals (ie, highest MADs or the most hazard categories classified per chemical) and “low variability” chemicals (ie, lowest MADs or only one hazard category classified per chemical). Chemical parameters such as functional use or chemical and physicochemical structural properties (ToxPrint and OPERA) could not account for the variability observed.

Accordingly, it is likely that variability among LD_50_ estimates for a single compound in our data set reflects biological variability inherent in animal models and/or the test method itself. It is important to note that although there are different iterations of the acute oral systemic toxicity test method with minor protocol differences (eg, up-and-down procedure, limit tests), they share key commonalities (species, dosing route, etc.), are all considered acceptable by regulatory authorities, and the results are used interchangeably for hazard and risk assessment purposes. Because this work is intended to support regulatory application, it was deemed appropriate to pool all obtained LD50 values despite the lack of experimental metadata. In the absence of complete study metadata, we were not able to consider known sources of biologic variability such as rat strain (Kacew and Festing, 1996; Walden and Schiller, 1985), sex (Fonsart *et al.*, 2008), age (Gaines and Linder, 1986), or vehicle. Such sources of variability have the potential to influence acute LD_50_ estimates across an order of magnitude and may explain some of the variability observed in our analyses. For example, the acute LD_50_ of 2,3,7,8-tetrachlorodibenzodioxin (TCDD) is 9.8–17.7 µg/kg in Long Evans rats, but is greater than 7200 µg/kg in Wistar Hannover rats (Pohjanvirta *et al.*, 1993). Similarly, sex differences can result in LD_50_ estimates that differ by several-fold between males and females. For example, female Sprague Dawley rats exhibit an oral LD_50_ for colchicine that is 2-fold lower than males (Wiesenfeld *et al.*, 2007), whereas the acute LD_50_ of 3,4-methylenedioxymethamphetamine is 2.4-fold lower in male Sprague Dawley rats compared with females (Fonsart *et al.*, 2008). In addition to strain and sex, rat age may also influence sensitivity toward a test chemical. The acute oral toxicity of 57 pesticides was compared across male and female adult or weanling Sherman rats, and LD_50_ estimates were significantly lower for adult rats compared with weanlings for 18 pesticides (up to 1.87-fold different; Gaines and Linder, 1986).

There are other factors not typically standardized across studies nor included in study metadata that may alter biological responses and potentially contribute to LD_50_ variability. Rodents are sensitive to their environment and previous work has shown that numerous factors can influence their biology, such as time of day/circadian cycle (Ede, 1973), and physical stressors such as restraint (Pearl *et al.*, 1966) or noise level (Lauer *et al.*, 2009), among others. For example, simply moving a rack of cages from the colony room into a new location, such as a procedure room, can induce a 12%–15% increase in thymus weight (Drozdowicz *et al.*, 1990), a 36% decrease in lymphocyte count (Drozdowicz *et al.*, 1990), or a 30%–40% increase in serum glucose in mice (Tabata *et al.*, 1998). Animal handling can also influence physiology; picking rats up by their tails rather than by their body can predispose them to convulsions (Everds *et al.*, 2013).

Rodent biology is sensitive to environmental factors but the potential for these stressors to impact acute oral LD_50_ estimates is not well characterized. There is evidence that stressors can directly impact key aspects of toxicology, such as alteration of stability or absorption of chemicals by changing gastrointestinal secretion or motility; disruption of vasoconstriction or vasodilation in different tissues, which affects distribution of chemicals; and impairment of metabolism of chemicals (Vogel, 1987, 1993). Standardized test methodology cannot feasibly encompass every single factor that may contribute to animal stress or otherwise serve as a potential source of variability. Given the numerous potential sources of variability in rodent studies, it is reasonable to conclude that even well-standardized test methods will not be able to control or account for every potential source of variability.

The curated data set compiled herein was used to establish a margin of uncertainty that could help in the assessment of NAMs. By bootstrapping MADs 5000 times, a representative MAD was obtained in order to compute a margin of uncertainty range of ±0.24 log_10_ (mg/kg). This result is within the same order of magnitude established in previous efforts such as Hoffmann *et al.* (2010), who noted that most of the 88 chemicals with rat data they evaluated had standard deviations less than 0.5 log_10_ (mg/kg) and calculated a median transformed standard deviation for all chemicals of 0.17 log_10_ (mg/kg). Recent work examining systemic effect levels from subacute, subchronic, chronic, multi-generation reproductive, and developmental toxicity studies found that unexplained variance ranged from 0.20 to 0.39 log_10_ (mg/kg), even after accounting for study protocol descriptors (Ly Pham *et al.*, 2020).

With the application of rat acute oral toxicity data for hazard category determination, it is important to understand reproducibility, how uncertain a hazard category may be, and the likelihood of repeated studies resulting in the same outcome. By applying the conditional probability analyses, it is apparent that specific potency ranges are more likely to be reproduced than others. This analysis was conducted using the same conditional probabilities approach as has been applied to ocular and dermal toxicity data to help identify which hazard categories’ determination may be no better than a coin toss (Luechtefeld *et al.*, 2016; Rooney *et al.*, 2021). Analyses conducted with the current data set suggest that acute oral toxicity in rats is also largely only 50%–60% reproducible for most hazard categories in both the EPA and GHS categorization schema based on percentages computed using the conditional probabilities approach on the expanded data sets comprising multiple studies per chemical to yield hazard category outcomes. This striking result is an important takeaway from this study to help characterize reproducibility of the existing *in vivo* study. Understanding the limitations and variance inherent in the method provides important context for subsequently using these *in vivo* data for training and evaluating alternative predictive methods.

Our analyses provide a global margin of uncertainty of ±0.24 log_10_ (mg/kg) characterizing the variability in the rat acute oral systemic toxicity study based on curated reference animal data. Applying this margin of uncertainty onto predictions made with alternative methods that predict rat acute oral LD_50_ values can serve as an approach to help identify acceptable LD_50_ ranges to target. For example, in the Collaborative Acute Toxicity Modeling Suite (CATMoS; Mansouri *et al.*, 2021), international predictive modeling efforts were undertaken to create consensus predictions for rat acute oral toxicity LD50s and hazard categories. To provide context to the predicted values in that modeling exercise, a 95% confidence interval allowed for the identification of outlier predictions and generation of consensus hazard category predictions. Such applications of this data set, or the margin of uncertainty generated herein, could substantially improve confidence in predicted values.

Understanding uncertainty and characterizing reference data helps provide much needed context to assess “gold standard” *in vivo* regulatory test methods. In fact, an ad hoc committee of the National Academies of Sciences, Engineering, and Medicine was recently charged with providing a review of the variability and relevance of existing laboratory mammalian toxicity tests for human health risk assessment to inform approaches for validation and establishing scientific confidence in new approaches. Herein we have compiled an extensive list of rat acute oral LD_50_ data from a large number of chemicals; we believe our extensive curation efforts allow these analyses to best represent the true variability of the acute oral lethality test. Experimental replication, chemical use, and chemical structure were not found to be sources of variability for disparate LD_50_ values arising from independently conducted acute oral toxicity tests. Finally, the margin of uncertainty derived herein can be leveraged to provide context for alternative approaches based on the understanding that the precision of NAMs that predict the LD_50_ is inherently limited by the precision of animal data.

## SUPPLEMENTARY DATA

Supplementary data are available at *Toxicological Sciences* online.

## Supplementary Material

kfac042_Supplementary_Data

## Notes

**Disclaimer:** This article may be the work product of an employee or group of employees of NIEHS, NIH, or other organizations. However, the statements, opinions or conclusions contained therein do not necessarily represent the statements, opinions, or conclusions of NIEHS, NIH, U.S. government, or other organizations. Integrated Laboratory Systems, LLC staff do not represent NIEHS, the National Toxicology Program, or the official positions of any federal agency.
