# Supplementary material for: Evaluation of Variability Across Rat Acute Oral Systemic Toxicity Studies
Source: Toxicol Sci. 2022 Apr 15;188(1):34–47. doi: 10.1093/toxsci/kfac042 (PMC9237992; doi:10.1093/toxsci/kfac042)
Supplement: kfac042_Supplementary_Data [file kfac042_supplementary_data.zip › kfac042-suppl_data/toxsci-21-0357-File009.docx]

**SUPPLEMENTARY FIGURES**

**FIGURE S1**


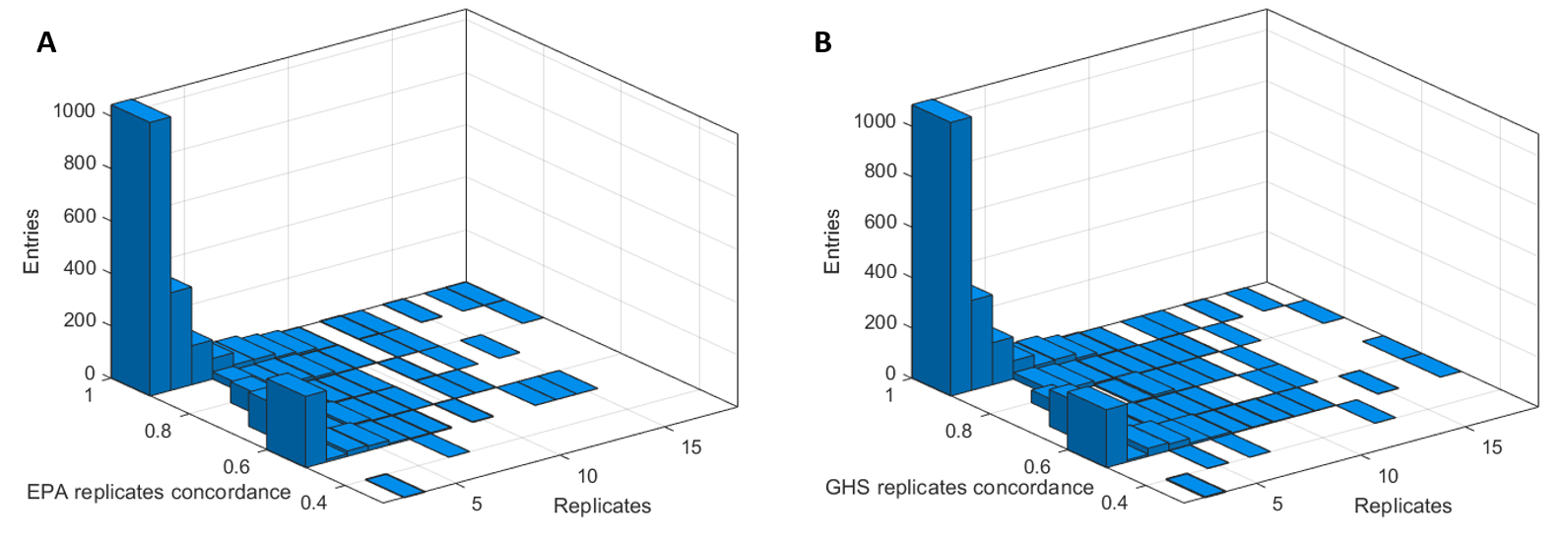


**Supplementary Figure S1.** Distribution of hazard categorization replicate concordance compared to replicate and entry counts. The expanded acute oral toxicity categorical inventory was evaluated for EPA category replicate concordance (A) and GHS category replicate concordance (B) relative to replicates per chemical and total number of entries in the inventory.
